# A novel ex vivo model for critical illness neuromyopathy using freshly resected human colon smooth muscle

**DOI:** 10.1038/s41598-021-03711-z

**Published:** 2021-12-20

**Authors:** Robert Patejdl, Felix Klawitter, Uwe Walter, Karim Zanaty, Frank Schwandner, Tina Sellmann, Katrin Porath, Johannes Ehler

**Affiliations:** 1 Oscar Langendorff Institute of Physiology, Rostock University Medical Center, Gertrudenstraße 9, 18057 Rostock, Germany; 2 Department of Anesthesiology and Intensive Care Medicine, Rostock University Medical Center, Rostock, Germany; 3 Department of General, Thoracic, Vascular and Transplantation Surgery, Rostock University Medical Center, Rostock, Germany; 4 Department of Neurology, Rostock University Medical Center, Rostock, Germany

**Keywords:** Neurological disorders, Experimental models of disease

## Abstract

Patients suffering from critical illness are at risk to develop critical illness neuromyopathy (CINM). The underlying pathophysiology is complex and controversial. A central question is whether soluble serum factors are involved in the pathogenesis of CINM. In this study, smooth muscle preparations obtained from the colon of patients undergoing elective surgery were used to investigate the effects of serum from critically ill patients. At the time of blood draw, CINM was assessed by clinical rating and electrophysiology. Muscle strips were incubated with serum of healthy controls or patients in organ baths and isometric force was measured. Fifteen samples from healthy controls and 98 from patients were studied. Ratios of responses to electric field stimulation (EFS) before and after incubation were 118% for serum from controls and 51% and 62% with serum from critically ill patients obtained at day 3 and 10 of critical illness, respectively (p = 0.003, One-Way-ANOVA). Responses to carbachol and high-K^+^ were equal between these groups. Ratios of post/pre-EFS responses correlated with less severe CINM. These results support the existence of pathogenic, i.e. neurotoxic factors in the serum of critically ill patients. Using human colon smooth muscle as a bioassay may facilitate their future molecular identification.

## Introduction

Critical Illness of any cause is associated with a wide range of neurological complications including neuropathy and myopathy in more than 80 or even up to 100 percent of patients, depending on the clinical definition applied^1,2^. The individual risk for critical illness neuromyopathy (CINM) is known to increase with several factors, e.g. the severity of disease, the need for mechanical ventilation and elevated blood glucose levels^3–7^. Additional intrinsic and environmental factors are likely to contribute, but many components of CINM-pathophysiology are yet largely unknown^8^. The definition of “Intensive Care Unit-Acquired Weakness” (ICU-AW) as a designation for muscular weakness of critically ill patients, that can be diagnosed solely by clinical examination and without electrophysiological characterization of the underlying alterations, has added additional complexity and even more questions to the field^9–11^.

Onset and worsening of neuromuscular disorder among patients receiving mechanical ventilation and sedation may be masked for days up to several weeks, showing its first manifestation long after its onset when patients suffer from complicated weaning, dysphagia and profound weakness^12,13^.

Although the relative contribution of cellular versus humoral factors in the pathogenesis of CINM is unknown, previous studies could identify soluble serum factors that are increased in patients suffering from CINM and ICU-AW^14–16^. However, most of them, e.g. cytokines and hormones, rather reflect the severity of critical illness itself and are of limited use for identifying individual patients that are prone to developing CINM or ICU-AW^2^.

A limitation of research on the pathogenesis of CINM and ICU-AW is the lack of experimental approaches for testing potentially harmful serum factors with regard to their effects on human peripheral nerve and muscle tissue. Specimen of human nerve-muscle preparations would be ideal bioassays to address this issue, avoiding potential inter-species effects that would inevitably weaken any conclusions obtained from studying human patient serum on animal preparations^17,18^.

Obtaining human (motor) nerve-muscle preparations of suitable size and quality from healthy volunteers to be used for bioassays is unjustifiable for ethical reasons. Therefore, it seems an attractive approach to consider materials that are more easily available, but yet allow measuring muscle contractions and synaptic transmission from neurons to muscle and their potential alteration by serum factors with presumed pathogenicity^19^.

Intestinal contractions are modulated by a complex network of neurons that are located between the longitudinal and circular smooth muscle layers. These neurons form synapses among each other and with the interstitial cells of Cajal (ICC) that form the interface between neurons and smooth muscle cells (SMC)^20^. Neurogenic contractions of isolated muscle from healthy regions of colonic segments obtained during routine surgery can be evoked in a stable manner for many hours when muscle strips are placed in organ baths under quasi-physiologic conditions^21,22^. The neurogenic contractions require physiological function of axons, neurons and synapses of enteric neurons. Despite the fact that the elementary processes of neuromuscular coupling in colonic smooth muscle are similar, but not identical to that in skeletal muscle up to the molecular level (some differences are outlined in Table 1), it may still serve as a valid bioassay to test detrimental effects of potentially noxious substances. To which degree the obtained results can be translated to skeletal muscle can only be clarified empirically by assessing correlations between alterations of smooth muscle and skeletal muscle function. In general, as is illustrated by the points addressed in Table 1, the intestinal muscle preparation contains an even broader spectrum of targets which contribute to its neurogenic responses, so it can be considered to be even more susceptible to noxious factors and thus may be a more sensitive bioassay.

**Table 1:** Selected differences between skeletal muscle cells, smooth muscle cells and their respective motoneurons.

Structures or parameters	Skeletal muscle	Intestinal smooth muscle
Discharge frequency	Rather high and modulated over wide range, e.g. around 40 Hz for full activation of upper extremity muscles^23^	Rather low and discontinuous, maximum around 10 Hz^24^
Nerve-muscle-interface	Direct: neuromuscular synapse (chemical) between motoaxon and muscle fiber^25,26^	Indirect: chemical synapse between motoneuron and ICC, electrical synapse between ICC and SMC^20,27,28^
Neurotransmitters	Acetylcholine, possibly modulated by glutamate (no human data)^29–31^	Acetylcholine, purines, and neuropeptides, e.g. substance P, complex local network with even more transmitters and mediators^22,32,33^
Muscle cell acetylcholine receptor	Nicotinic receptor (α_1_)_2_β_1_δε^34^	Muscarinic receptors (M_2,_ M_3_), nicotinic receptors of α3β2-, α3β4- and/or α7-subtype^35,36^
neuronal action potential	carried by sodium channels, most likely NaV 1.6^37,38^	carried by sodium channels, most likely NaV 1.5, NaV 1.9^39^

The aim of this pilot study was to establish a bioassay that is based upon the neurogenic and pharmacological responses of human smooth muscle obtained from regular colon tissue of patients undergoing elective surgery. Colon smooth muscle strips were incubated with serum collected from controls and critically ill patients at the 3rd and 10th day of illness. The resulting changes in neurogenic and pharmacological responses were compared between controls and patients with- and without neuromuscular alterations as defined by clinical and electrophysiological testing. It was of special interest whether the pattern of functional changes seen on the level of muscle strips exposed to serum drawn at an early stage of disease would be related to the patients’ neuromuscular condition in the later course of disease.

## Results

### General characteristics of critically ill patients and colon tissue donors

The screening and recruitment process which is described in detail in the methods—section finally allowed the inclusion of 48 critically ill patients for further analysis. For all of them, a sequential organ failure assessment (SOFA) score of ≥ 8 had been stated on ≥ 3 days after admission to the intensive care unit (ICU). Six-hour organ bath experiments studying the effects of these patients’ sera on colon smooth muscle strips could be completed for 43 sera obtained at day 3 of disease and for 38 sera obtained at day 10. Additionally, 15 experiments were performed with sera from healthy controls. Reasons why patient sera could not be studied were failure for sample collection due to logistic reasons (8 sera) or technical problems that occurred within the experimental procedure itself: Five strips ruptured during serum incubation or subsequent activations and two were displaced from their original position relative to the stimulation electrodes.

Patients’ mean age was 67.1 ± 14.4 years and 29 were females. Among the 15 healthy subjects who donated blood for control serum incubation experiments, there were 6 females. The mean age of the control serum donors was 55.9 ± 10.6 years and thus younger than that of the patient group (p < 0.01).

The 25 colon tissue donors had a mean age of 74.6 ± 9.4 years (11 females).

The primary diseases which led to the complication of critical illness in the serum donor group were coronary or aortic disease (28), gastrointestinal disease (6), multiple trauma (4) or other conditions associated with septic shock or hemorrhage following surgery (7). Among the 26 tissue donors, 16 patients had a diagnosis of colorectal carcinoma, 4 of rectal prolapse, 4 of sigmoid diverticular disease and 2 of rectocele. In case of the samples from carcinoma-patients, the histopathology of the resected colon segments from which the material used in this study had been taken confirmed that they were free of tumor cells.

### Neuromuscular alterations and clinical parameters of critically ill patients

**Figure 1:**
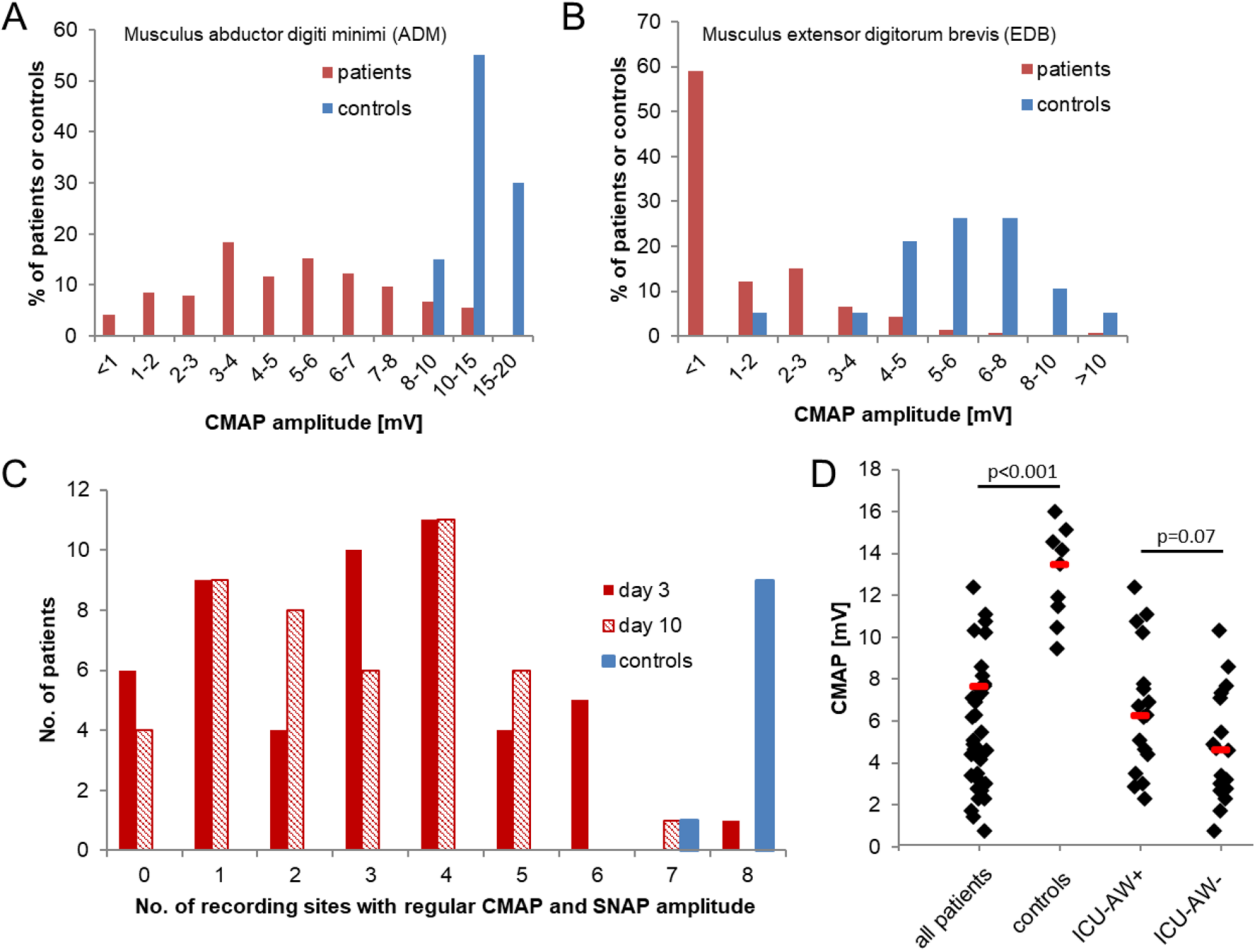
Electrophysiological parameters of neuromuscular function of patients and controls. (**A**) Distribution of CMAP-amplitudes recorded from the upper extremity (ADM, bilateral) among patients and controls. (**B**) Distribution of CMAP-amplitudes recorded from the lower extremity (EDM, bilateral). (**C**) The number of recording sites giving SNAPs or CMAPs of regular amplitude was summed up for each patient. The distribution of patients or controls according to their individual sum score is displayed. For patients, separate distributions are shown based upon the measurements done at the 3rd and on the 10th day of their critical illness. (**D**) ADM-CMAPs recorded at day 10 and averaged from both sides in patients with and w/o ICU-AW. Red bars depict mean values. Differences between all patients and all healthy controls and differences between ICUAW + /− subjects were separately tested with t-tests for independent samples.

Compared to the 43 patients whose nerves and muscles were studied, the amplitudes of compound muscle action potentials (CMAP) were significantly higher in healthy controls (Fig. 1D). Furthermore, irrespective of the time point of measurement, CMAP-amplitudes obtained from the Musculus abductor digit minimi (ADM) were larger than from the Musculus extensor digitorum brevis (EDB) in both patients and controls (Fig. 1A,B). Taken together for both left and right side and both time points, 103 of the 173 detectable ADM-CMAPs in patients (59.5%) had physiological amplitudes which are defined as CMAP > 4 mV (Fig. 1A). By contrast, EDB-amplitudes were below 4 mV and thus pathologic according to the aforementioned definition in 97% of patients (Fig. 1B). Of the 10 healthy controls, only one had a sural sensory nerve action potential (SNAP)-reduction and one a reduced CMAP of both EDBs. The findings of the complete electrophysiological examination were regular in only one patient at day 3 and in none at day 10. The overall-number of unaltered recording sites in patients did not differ between the time points of examination (Fig. 1C).

The concept of a solely clinical diagnosis of critical illness—related neuromuscular complications (ICU-AW) did not prove useful in the studied patient sample: the defining muscle research council sum score (MRCC-SS) could be assessed in only 17 patients at day 3 and in 35 at day 10. Of these, seven and 17, respectively scored < 48 and could thus be defined to suffer from ICU-AW according to the definition suggested by Stevens^40^. However, the amplitudes of ADM—CMAP recorded at day 10 did not significantly differ with respect to the patients’ ICU-AW-status (Fig. 1D; p = 0.49).

After day ten, the clinical diagnosis of CINM was stated in the 18 patients with MRC-SS scores below 48 and in seven other patients according to the criteria given in the methods-section, i.e. based upon their MRC-SS plus a potential loss of muscle reflexes and reductions of SNAP and CMAP-amplitudes at 4 or more recording sites studied in electroneurography (ENG). Basic clinical and laboratory data of the resulting groups are given in Table 2. The laboratory parameters included were measured from samples obtained during the same blood draw at which the serum for organ bath experiments was taken. Significant group differences were only seen for the SOFA-scores at day 3 and 10.

**Table 2:** Clinical and laboratory characteristics of patients according to their ICU-AW status at day 10 of their critical illness; p-values that indicate significant differences are printed in bold.

	CINM−	CINM+	p-value
Patient no.	18	25	
mean age (years ± SD)	67.89 ± 14.7	66.6 ± 13.82	0.770
sex (% male)	0.72	0.64	0.570
mean APACHE II ± SD	25.11 ± 4.78	25.72 ± 6.13	0.727
SOFA ± SD day 3	10.28 ± 2.49	13.12 ± 2.86	**0.002**
SOFA ± SD day 10	4 ± 3.88	7.63 ± 4.13	**0.006**
% diabetes*	56	84	**0.041**
pH	7.42 ± 0.05	7.42 ± 0.07	0.854
K^+^ (mM)	4.31 ± 0.5	4.5 ± 0.45	0.199
Na^+^ (mM)	143.33 ± 3.68	144.16 ± 4.35	0.516
Ca^2+^ (mM)	1.16 ± 0.06	1.14 ± 0.07	0.596
Glucose (mM)	6.92 ± 1.3	7.64 ± 1.86	0.164
CRP (mg/l)	214.94 ± 143.32	231.42 ± 116.65	0.691
PCT (ng/ml)	5.02 ± 8.96	11.79 ± 21.13	0.209

	Healthy controls	Patients day 3	
CMAP ADM (mV)	13.3 ± 2.3	4.9 ± 2.2	**< 0.0001**
CMAP EDB (mV)	6.1 ± 2	1.6 ± 1.5	**< 0.0001**
SNAP radial nerve (µV)	41.7 ± 13.1	12.7 ± 6.6	**< 0.0001**
SNAP sural nerve (µV)	16.2 ± 4.3	11.8 ± 5.5	**0.04**

	Healthy controls	Patients day 10	
CMAP ADM (mV)	13.3 ± 2.3	5.5 ± 2.9	**< 0.0001**
CMAP EDB (mV)	6.1 ± 2	1.2 ± 1.3	**< 0.0001**
SNAP radial nerve (µV)	41.7 ± 13.1	14.2 ± 6.2	**< 0.0001**
SNAP sural nerve (µV)	16.2 ± 4.3	8.8 ± 5.1	**< 0.001**

Demographic and clinical parameters except for the SOFA-score refer to the day of admission, laboratory parameters refer to day 3. For numerical data, p-values were calculated using t-tests for independent samples. For categorical data, χ^2^ tests were used.

*Only patients without manifest diabetic polyneuropathy were included in this study.

### Contractile responses of muscle strips

**Figure 2:**
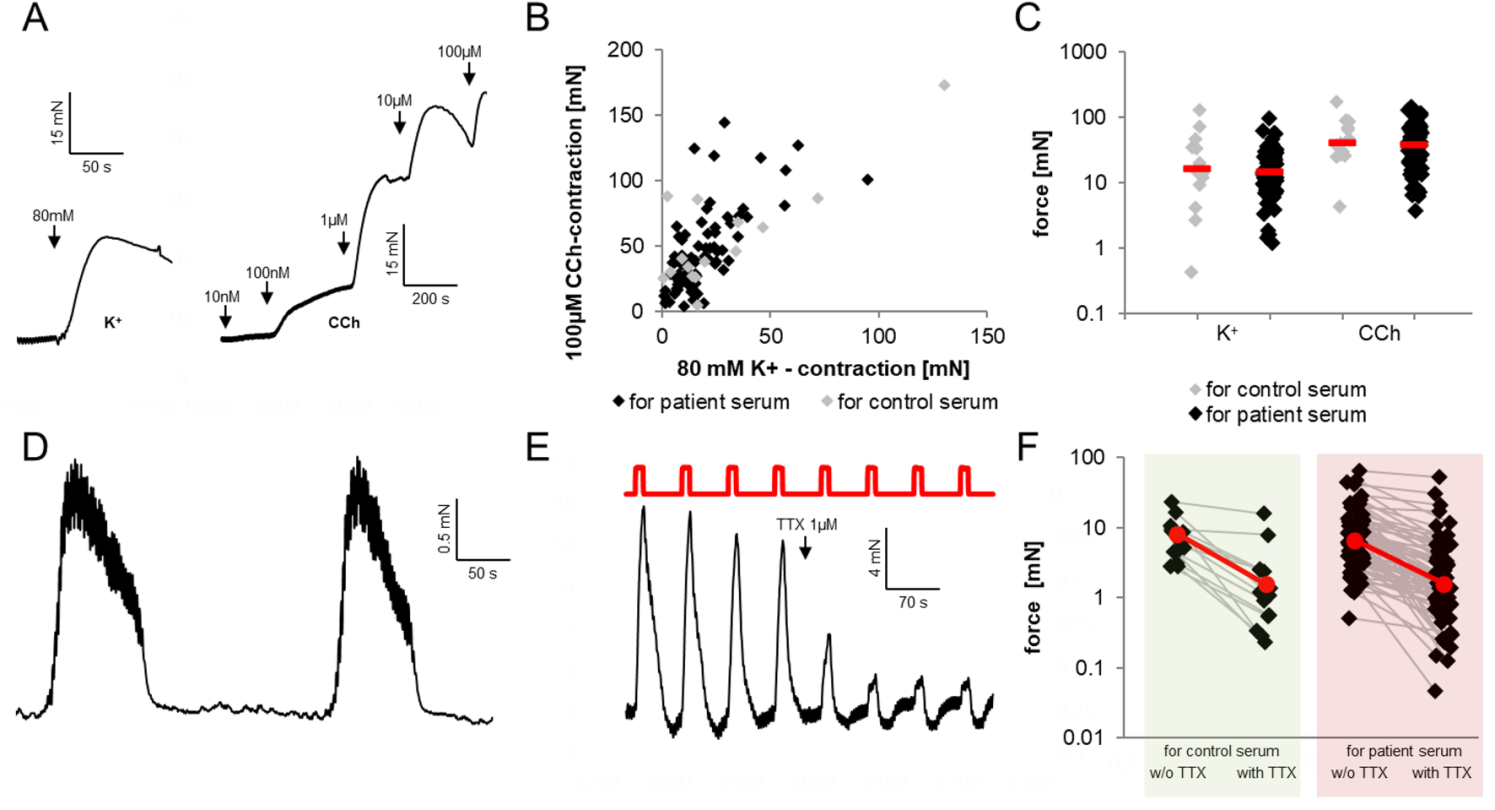
Representative traces of spontaneous mechanical activity and responses of human colonic smooth muscle strips to high-K^+^, CCh and electric field stimulation. (**A**) Contractile response to an increase in K^+^ and cumulative CCh applications in the organ bath. Arrows depict the time point of substance application. (**B**) Correlation of responses to the highest CCh-concentration and high-K^+^ within strips shown in grey for strips later on incubated with serum from healthy controls and in black for those later on incubated with patient’s sera. (**C**) Peak amplitudes of mechanical responses to high-K^+^ and CCh, again separated according to the type of serum used for the subsequent incubation period. (**D**) Spontaneous rhythmic activity of a muscle strip exhibiting low-amplitude, high-frequency waves superimposed on low-frequency-high-amplitude contractions. The amplitude of ripples was measured using the “mean cyclic height”-function in LabChart over a representative 10-s period. Low-frequency waves occurred in 24, ripples in 65 of 98 muscle strips. (**E**) Rhythmic application of trains of electric field stimulations (red trace depicts timing of trains) induces regular contractions of colonic smooth muscle strips (black trace). Application of tetrodotoxin (TTX) leads to a marked reduction in the amplitude of responses. Note the spontaneous high-frequency, low-amplitude activity underlying the pattern of exogenously evoked contractions. (**F**) Effects of 1 µM TTX on EFS-triggered contractions prior to serum incubation in muscle strips for later incubation with control or patient serum, respectively. Red rhombs and bars depict median values.

Following the equilibration period, contractions could effectively be evoked by adding potassium ions (K^+^) or the muscarinic agonist carbachol (CCh) to the organ bath (Fig. 2A). Whereas K^+^ activates all cells directly via global depolarization, CCh activates SMC directly and indirectly by stimulating the ICC-network via muscarinic receptor activation. The absolute amplitude of responses can be used as an estimate of the number of vital cells that are contained in the tissue strips. Its large variation reflects differences in muscle layer thickness and in surgery- and preparation related stress. Despite this considerable variation, there was a significant correlation between the maximum amplitudes of high-K^+^ and CCh responses in individual muscle strips before incubation with serum from patients and controls (strips used for control sera: r = 0.82; strips used for patients’ sera: r = 0.68, Pearson’s correlation coefficient, p < 0.01 for both). Neither the intrinsic correlations between CCh- and K^+^-responses nor the grouped response amplitudes for both stimuli differed between strips that were later on used for testing serum from controls nor patients (Fig. 2B,C).

In parallel to the responses evoked by substance application, spontaneous phasic contractions occurred in the majority of strips (74/98) over the time of serum incubation and were composed of high-frequency, low-amplitude activity (“ripples”) with superimposed low-frequency, high-amplitude contractions (Fig. 2D). The amplitudes of both varied between muscle strips and over the time of measurements. In the 39 preparations in which ripples were present before and after serum incubation, their amplitude declined significantly over the time of serum incubation [pre: median (IQR) 0.83 (0.39–2.23) mN; post: 0.67 (0.33–1.49) mN; p = 0.017, Wilcoxon-rank-sign-test]. High-amplitude contractions occurred in 24 of the 98 preparations by the end of the experiments. In contrast to ripples, their amplitudes and frequencies over time were so variable overtime that statistical processing was omitted for them. We could find no correlations between changes in phasic activity and clinical parameters of the serum donors or the source of serum (healthy controls vs. patients day 3 vs. patients day 10).

The application of EFS resulted in reproducible contractile responses which were of the “on”-type in the majority of specimen (82/98), meaning that the force increase appeared already while electrical stimuli were applied and not after the end of stimulation (“off”-type response, the background of on/off-contractions is addressed by Aulí et al.^22^). Tetrodotoxin (TTX, 1 µM) applied before serum incubation inhibited the responses of muscle strips to EFS (Fig. 2E). The amplitudes of EFS-induced contractions varied over a wide range in between muscle strips, but the inhibitory effect of TTX did not differ between muscle strips which were later on incubated in serum from healthy controls and those that were exposed to patient serum (Fig. 2F).

**Figure 3:**
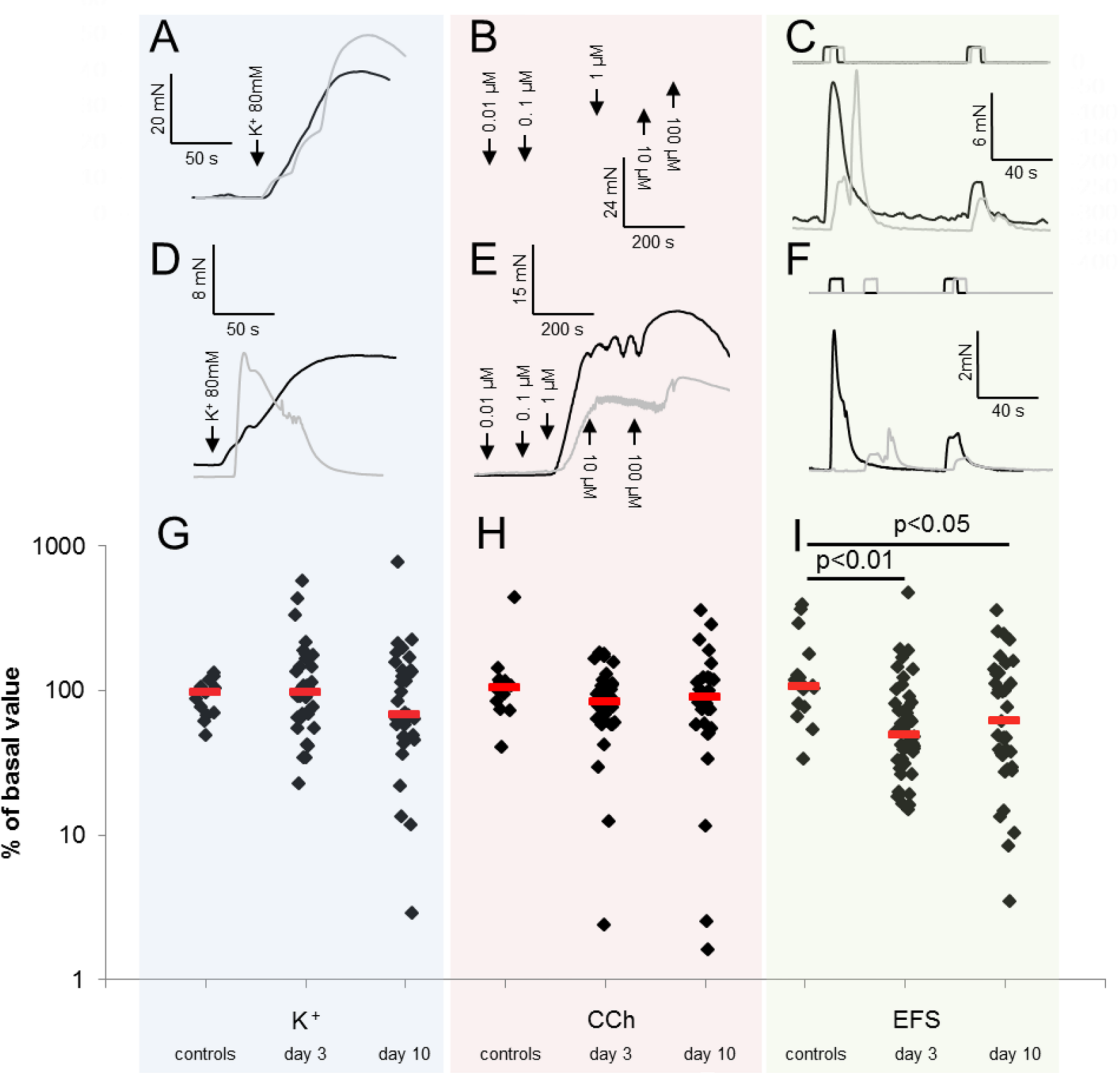
Contractile responses to high-K^+^, CCh and EFS before and after 3-h of incubation with human serum. Black traces: response prior to serum incubation; grey traces: response at the end of three hours serum incubation. Between the subsequent EFS depicted in (**C**), 1 µM TTX was applied. (**A**–**C**) representative original traces from strips incubated with serum obtained from a healthy control subject; (**D**–**E**) traces from strips incubated with patient serum obtained at day 3 of critical illness. Applied stimuli were high-K^+^ (**A**, **D**), CCh (**B**, **E**) and EFS (**C**, **F**); (**G**–**I**) data and median values of contractions evoked from strips incubated either with serum from healthy controls or with serum obtained from patients at day 3 or at day 10 of their critical illness; stimulations were high-K^+^ (**G**), maximum concentration of CCh-staircase (**H**) and EFS (**I**). Numbers over horizontal lines in (**I**) designate significance of differences as calculated by one-way ANOVA with Dunn’s post-test.

After the 3-h period of incubation with serum from patients or controls, responses to EFS, CCh and high-K^+^ could still be detected in all tested muscle strips (Fig. 3). Regarding the changes in shape and time course of responses during serum incubation, there were no significant differences related to the origin of sera, but variance between muscle strips was high, especially regarding the shape of K^+^ responses (compare Fig. 3A and D). In accordance to Fig. 2F, the reduction of EFS-responses by TTX can be observed between the subsequent EFS-responses displayed in Fig. 3C and F. In those strips incubated with serum from healthy controls, the amplitudes of responses to all types of stimuli did not change significantly over time.

In strips incubated with patient serum obtained at day 3 and day 10 of disease, responses to EFS decreased significantly. When serum from healthy controls was used instead, no change in EFS could be detected (Fig. 3I). As in the case of K^+^ and CCh, the EFS-response amplitude after serum incubation was divided by that seen before serum incubation. The ratios obtained by this procedure, given here as %-values, were then compared according to the different source of sera. (The resulting median ratios were: 106% for serum from healthy controls; 49% for serum obtained at day 3 and 62% for serum obtained on day 10 of illness. One-way-ANOVA on ranks detected a difference between the three serum-groups (p = 0.003). Dunn’s groupwise comparison confirmed differences between the effects of serum from healthy controls and that obtained at day 3 (p < 0.01) and day 10 (p < 0.05), but there was no difference between effects of sera obtained at day 3 and 10 when compared with each other.

Responses to high-K^+^ and CCh were compared analogously and did not change significantly over time in strips exposed to patient serum, neither regarding the amplitude of responses (Fig. 3G,H) nor the concentration-dependency (CCh EC_50_ after serum incubation, median (IQR): healthy control serum: 1.4 (0.7–3.8) µM; patient serum, day 3: 1.7 (0.8–6.8) µM; patient serum, day 10: 1.5 (0.7–22) µM, One-Way ANOVA: p = 0.44).

**Figure 4:**
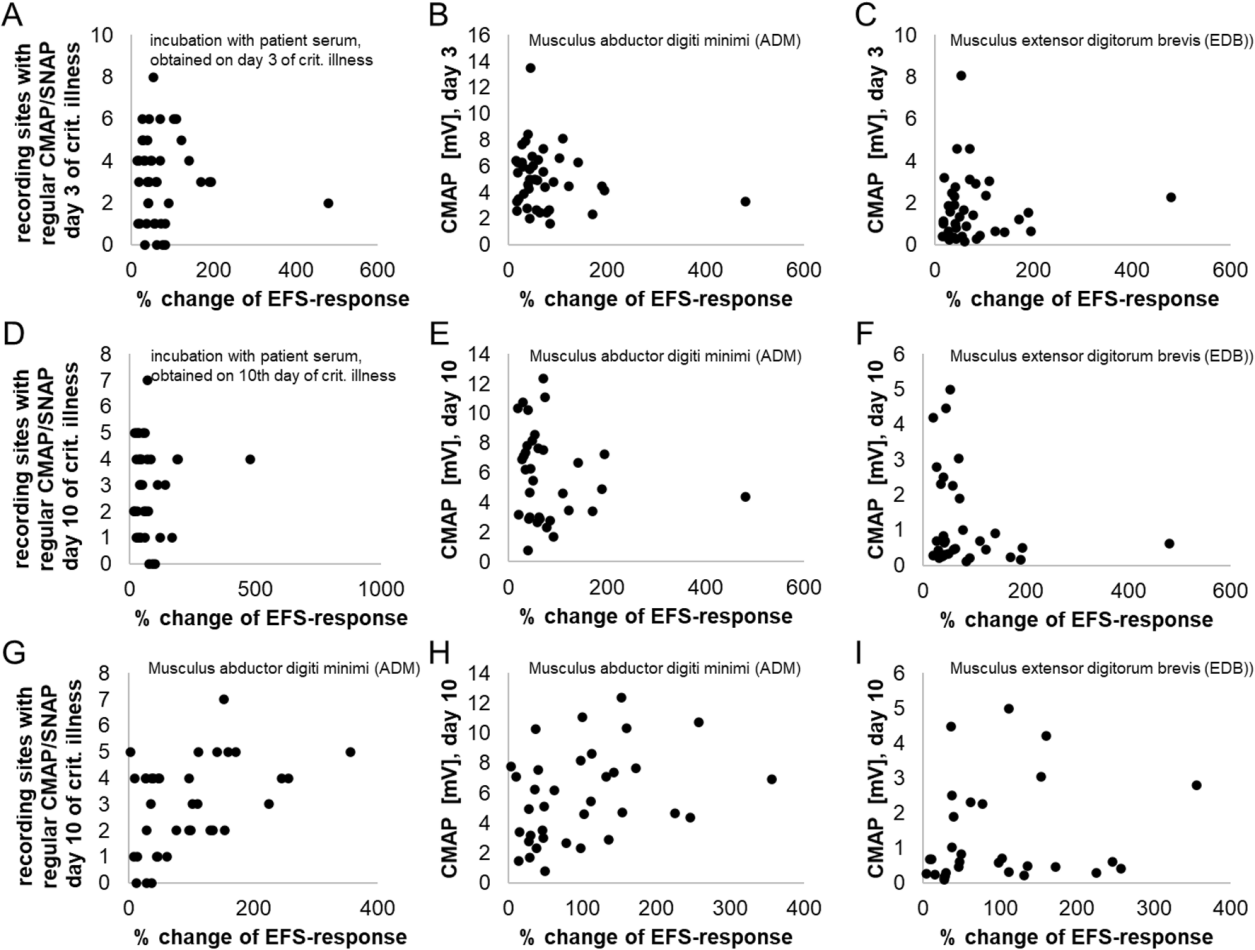
Correlations (Spearman’s rank correlation) between clinical electrophysiological findings in critically ill patients and changes of electric field stimulation responses of colon smooth muscle strips incubated with these patient’s sera; (**A**–**C**) Sera obtained at day 3 of critical illness, related to electrophysiological parameters on day 3. (**D**–**F**) Sera obtained at day 3 of critical illness, related to electrophysiological parameters on day 10. (**G**–**I**) Sera obtained at day 10 of critical illness, related to electrophysiological parameters on day 10. (**B**, **E**, **H**) Mean CMAP amplitudes of upper extremity (ADM) muscles. (**C**, **F**, **I**) Mean CMAP amplitudes of lower extremity (EDB) muscles; significant correlations are present in G (ρ: 0.38; p = 0.021) and H (ρ: 0.37; p = 0.047).

The observed changes in the EFS-responses were tested for associations with the patients’ clinical and electrophysiological findings. The observed changes in EFS-responses of colon strips exposed to patient sera obtained at an early stage of disease were not related to these patients’ electrophysiological alterations, neither in terms of the number of affected recording sites nor regarding the CMAP-amplitudes from upper- and lower extremities (Fig. 4A–C). The changes in EFS-responses of smooth muscle strips induced by serum obtained at day 3 were also unrelated to the electrophysiological alterations seen in the individual serum-donors at day 10 (Fig. 4D–F). In contrast, changes in EFS-responses occurring during incubation with sera obtained at day 10 of critical illness showed a significant correlation with the number of unaltered recording sites (SNAPs and CMAPs) and with the amplitude of ADM-CMAPs, but not with EDB-CMAPs (Fig. 4G–I). The correlation was positive for both, with increases in EFS-responses occurring when smooth muscle strips were incubated with sera from patients with less electrophysiological alterations. Partial correlation coefficients were calculated based upon the bivariate Spearman-ρ-coefficients and gave no evidence that the association between the number of unaltered nerves and patient’s serum effects on EFS were confounded by patients’ age or systemic inflammation (partial ρ, corrected for age: 0.38; partial ρ, corrected for CRP: 0.39; partial ρ, corrected for PCT: 0.41).

Sera obtained at day 3 from patients classified as CINM + altered EFS-responses during the incubation period in a similar manner as those obtained from CINM- patients [median change in EFS—response(IQR)%: CINM+: 48.6 (28.7–72.5)%; CINM−: 53.6 (37.4–70.6); p = 0.98]. Likewise, sera from day 10 caused no changes in EFS-responses related to CINM-status [median change in EFS—response ± IQR: CINM+: 77.5 (27.7–116.7)%; CINM−: 47.6 (38–202.6)%; p = 0.46].

## Discussion

The findings of this study support the hypothesis that humoral factors may be involved in the pathogenesis of CINM which, in line with previous studies, was a frequent complication of critical illness in the studied patient sample^41–46^. Despite the potential advantages of using pragmatic clinical approaches and diagnoses as ICU-AW to assess patients for neuromuscular dysfunction, samples of patients with this diagnosis are likely to contain patients with biologically distinct diseases with predominant neuropathy, myopathy or immobilization atrophy^10^. While specific alterations in nerve conduction studies can be assumed to reflect distinct pathophysiological processes at defined anatomical structures, changes in clinical scores (e.g. MRC-SS) may be not applicable in patients with impaired consciousness, may be confounded by various other factors and, even worse, may be subject to a rather high inter-rater variability, making them less suitable to serve as readouts of disease severity for studies on biomarkers of any kind^46–48^.

A major point of discussion in the pathophysiology of generalized neuromuscular dysfunction in critically ill patients is whether alterations of nerves and skeletal muscles are caused by soluble humoral factors or by cellular cytotoxicity^2,49^.

The results of the present study give first evidence that critically ill patients’ serum contains factors which alter the function of human neurons in their native tissue environment. To ensure the neurogenic nature of the EFS-induced contractions analyzed in this study, stimulus frequencies were kept within the physiological range of enteric motor neuron discharges^24^. Furthermore, TTX-sensitivity of the responses was tested twice during in each experiment. Finally, the shape of EFS-responses was analyzed and showed the typical pattern of dominance of “on” over “off”-type contractions, reflecting a dominance of excitatory transmitter release with the applied stimulus paradigm which has been described in the literature^22^. Stable neurogenic contractions could be elicited over more than three hours of incubation time when sera of healthy volunteers were used. The magnitude of the obtained EFS responses was lower than high-K^+^ or CCh contractions, the relation between the different responses being comparable to that we observed in murine fundus preparations in a recent study using the same setup and equivalent EFS-parameters^50^. The most likely explanation is that EFS-pulses, inevitably exciting all kinds of intramural nerve fibers, activate the coincident release of excitatory and inhibitory transmitters which leads to dampened response compared to purely excitatory transmitter actions exerted by CCh or direct SMC-depolarizations by K^+^. This is illustrated by the fact that contributions of specific transmitters to the summative contraction can be dissected by using specific receptor antagonists or by pre-activation with the dominant excitatory neurotransmitter (mostly acetylcholine), as has been demonstrated for instance by Windscheid et al.^51^. Another factor that is likely to contribute to the difference in response amplitudes is that with the used type of stimulation electrodes, only a restricted area of tissue is reached by EFS, whereas the exogenously applied substances CCh and K^+^ likewise reach all contractile cells within the muscle strip. Importantly, the amplitudes of contractions evoked by direct activations of smooth muscle cells using high-K^+^ or CCh remained stable over time, too. Thus, it can be concluded that serum incubation did not cause changes in the excitability or contractility of smooth muscle cells and that changes in EFS-triggered contractions indeed are reflecting alterations of neuronal function or neurotransmission.

In contrast to their stability after incubation with serum from healthy controls, EFS-responses were significantly reduced following incubation with serum from critically ill patients. Since again neither responses to high-K^+^ nor to CCh were altered, an interference with neuronal function can be assumed. An alternative explanation would be an affection of interstitial cells of Cajal (ICC) which are considered to be the primary target of both excitatory and inhibitory motor innervation in the colon^20,52^. Since spontaneous rhythmic contractions are only slightly reduced following patient serum incubation, at least strong effects on the ICC-subpopulations responsible for pacemaking seem unlikely. Furthermore, ICC are considered to be the primary targets of muscarinic agonists, enforcing contractions by passing their increased pacemaker currents to adjacent smooth muscle cells via gap junctions. As neither the EC_50_ of CCh nor the maximum response amplitudes to CCh differ between strips incubated with patient or control serum, altered ICC-function is even more unlikely to be the cause of the observed changes in EFS-responses.

Although the median reduction of EFS-responses observed in strips incubated with patient serum did not differ between sera obtained at day 3 or day 10, the variance of individual values was larger when strips were exposed to serum from day 10 (Fig. 3I). This increase in variance might be suggestive of an additional, yet unknown neurotoxic substance which is present in serum obtained at day 10 at highly variable concentrations. From our point of view, this assumption is strongly supported by the observed correlation between the observed effects of individual sera on EFS with the extent of neurophysiological alterations present in the patients from whom these individual serum samples had been obtained (Fig. 4G).

Some limitations of the study need to be considered. The complex diagnostic workup at two different time points and the decision to include only patients with strictly defined severe illness and without any preexisting neuromuscular disease within a minimum duration of three days after the onset of critical illness prevented the inclusion of a larger number of patients. Therefore, the power of this study might have been insufficient to detect rather subtle effects of sera, e.g. on SMC responses to exogenous stimuli. Furthermore, the logistic complexity and duration of the organ bath experiments led to a drop-out of some patients, which might have confounded the results. Another potential bias, though highly unlikely from our point of view, is that ruptures of smooth muscle strips might have been related to disease-related serum effects. Intestinal perforations have been reported as complications of extremely severe motility disorders in critically ill patients, Furthermore, a recent study gave evidence from histopathology for critical illness induced damage of the intestinal wall, including the tunica muscularis^53,54^.

Finally, it has to be emphasized that the observed correlations between serum effects on smooth muscle strips and patients’ electrophysiological parameters is not necessarily indicative of a causal relationship between serum factors and neuromuscular dysfunction. Our observations might instead point to other, yet unknown alterations of serum composition which might influence smooth muscle function. Although we did not observe relevant differences in pH, electrolytes and glucose concentration between the groups, we cannot exclude that other parameters might have caused the observed biological effects. Finally, it is of interest to consider the fast onset of effects that was observed within the rather short incubation duration applied in our experiments with the permanent exposition of the patient’s tissue with the potentials neurotoxic factors. One might speculate that metabolic stress and damage to which the colonic tissues have been exposed during transport and preparation might accelerate the deleterious effects of potential noxious substances to which the specimen are exposed during serum incubation. Although conditions in organ bath experiments are designed to mimic physiologic conditions, especially the neuronal structures located in the deep myenteric plexus are likely to suffer from latent hypoxia and metabolic stress in the absence of physiological circulation.

Our data revealed a correlation between the level of neuromuscular alterations at day ten and the effects of serum obtained at the same time in the ex vivo experiments. There was no correlation between the effects of serum taken on day 3 and the neuromuscular impairment at that time, most likely due to the fact that serum effects on EFS-responses were in a rather narrow range. Together with the observation of the severely altered neuromuscular function at day 3, it seems likely that the neurotoxic components were present in a higher proportion in serum samples obtained at this early time point of critical illness, which may be supported by the trend towards a stronger reduction of EFS-responses observed with serum from day 3 when compared to serum from day 10 and the evident heteroscedasticity between both groups [median reduction (IQR)%: day 3: 51 (33–83)%; day 10: 62 (34–132)%, p = 0.44].

Numerous studies have demonstrated that changes in serum concentrations of various endogenous metabolites and mediators accompany the onset and later course of CINM, but it has proven difficult to assign the distinct contribution of systemic inflammation, general disease severity and organ failure^2^. Elucidating the contribution of single factors is complicated by the overlap between central nervous system complications of critical illness (e.g. delirium) with highly variable degrees of polyneuropathy and myopathy in a context of polypharmacy and altered metabolism of the critically ill. The experimental model introduced here offers the possibility for a wide range of studies and analyses to clarify the contribution of single serum components or defined exogenous substances on neurons and other cell types within their native microenvironment.

Two major advantages result from the introduced approach of using human instead of animal tissues. First, issues of interspecies-variations and potential immunological reactions can be avoided. Second, there is no need to generate animal models, which is in line with the commitment to animal welfare and the 3R (reduce, replace, refine)—principle. From our point of view, this seems reasonable since numerous molecular components which are considered to be affected in CINMCINMare also present and functionally relevant in the enteric nervous system and smooth muscle: Besides differences in the molecular subtypes and the contribution of nicotinic vs. muscarinic receptors, many other key elements of neuronal function, e.g. voltage sensitive ion channels for sodium, potassium and calcium, ion exchangers and pumps as well as many other neuron-specific proteins are expressed in both tissues^19^. The more complex arrangement of neurons in colonic tissue including its interneuronal synapses and the multitude of neurotransmitter systems offers even more targets for neurotoxic compounds than nerve-skeletal muscle preparations would. For future studies, modifying or extending the bioassay with serum incubation of cultured human tissues might offer an interesting option to learn more about the pathophysiology of the effects seen in our experiments and to reduce the logistic complexity of the approach.

Taken together, the results of this study give evidence that the neuronal circuits and the contractile apparatus of colonic smooth muscle may indeed serve as a suitable bioassay or ex vivo model for CINM and the underlying pathophysiological changes.

## Methods

This prospective study was conducted in accordance with the Declaration of Helsinki. It has been approved by the local ethics committee of the University of Rostock (A 2016–0016) and is registered in an international database (ClinicalTrials.gov: NCT02706314). All patients and controls or their legal representatives gave their consent and all patient-related procedures. Muscle ultrasound and serum parameters as well as gastrointestinal complications from subsets of the study cohort were published previously^14,55^. The experimenters conducting the organ bath experiments were blinded to the clinical classification of patients as ICU-AW or CINM positive or negative.

### Study population and patient selection

We screened a total of 7912 patients ≥ 18 years of age admitted to the Department of Anesthesiology and Intensive Care Medicine at the University Medical Center Rostock between 10/2016 and 12/2018. Of these, 1819 had a SOFA score ≥ 8 on ≥ 3 consecutive days within the first 5 days after ICU-admission and were therefore defined to be critically ill. Patients were excluded if they had a known history of any neuromuscular disease including diabetic polyneuropathy, had received high-dose glucocorticoid treatment (i.e., more than 300 mg of methylprednisolone or equivalent doses of other steroids) in the course of treatment or if they had been treated at another ICU for more than 24 h. Furthermore, patients from whom informed consent could neither be taken directly or by their legal representatives were not included, as well as patients who participated in other clinical trials or who were expected to die within 24 h.

### Patient serum collection

On day 3 and 10 after ICU-admission, blood was drawn from each patient of the study cohort. Serum tubes containing a total of 40 ml blood were transferred to the department of physiology, centrifuged at 2000×*g* for 10 min at 4 °C (Eppendorf centrifuge 5702 RH, Germany), transferred to 50 ml plastic tubes (Sarstedt, Germany) and stored at – 80 °C. In parallel, pH, glucose and serum concentrations of K^+^, Na^+^ and Ca^2+^ were estimated by point-of-care testing (Radiometer ABL90 Flex, Radiometer GmbH, Krefeld, Germany).

### Electrophysiology and clinical assessment of neuromuscular function

Motor and sensory ENG were performed on day 3 and day 10 of disease. Compound motor action potentials were recorded from the ADM and the EDB. Sensory nerve action potentials (SNAP) of the radial and sural nerves where obtained using the antidromic technique. The minimum amplitudes for normal responses were defined to be at least 4 mV for CMAPs and 7.5 μV for the radial and the sural nerves. Furthermore, a standardized clinical assessment of the patient’s neuromuscular function was performed. The MRC-SS was assessed on day 3 and 10 and ICU-AW was diagnosed in patients achieving < 48 points^5^. A sedation holiday of at least 2 h before examination was performed in all patients with mechanical ventilation and continuous analgosedation. When patients were not eligible for MRC-SS assessment, neurological examination and second ENG were considered and CINM status was assigned when all of the following criteria applied: muscle reflexes were absent, muscle tone and movements induced by painful stimuli were reduced and electrophysiology showed reduced SNAPs and CMAPs at 4 or more recording sites. Furthermore, conduction velocities were calculated in all stated nerves to exclude pre-existing or acute demyelinating diseases.

### Preparation of colon smooth muscle strips

Informed consent for the use of tissue for experimental research was obtained from patients ≥ 18 years of age undergoing elective partial colectomy for the indications reported in the results-section. Patients with any neurological disease, chronic inflammatory bowel disease or prior radiation- or chemotherapy, intestinal ischemia or recent corticosteroid-treatment were not included. Immediately after extirpation, a ring segment of 2–3 cm length of the resected colon segment was transferred in a vessel containing 500 ml of preparation buffer that had been cooled to 4 °C. Within less than one hour, the vessel was brought to the laboratory. The tissue ring was then pinned into petri-dishes laid-out with silicone and filled with fresh cooled preparation buffer, cut open along and cleaned by repeated flushing. The mucosa was then dissected from the tunica muscularis which was subsequently pinned and slightly stretched in the longitudinal direction. Under the dissection microscope (Olympus SZ40, Olympus, Shinjuku, Japan), longitudinal strips of tissue of 2 cm length and 1.5–2 mm width were then cut and placed into a separate vessel with cooled preparation buffer. Within the subsequent 24 h, the tissue strips were then tethered to plastic holders with embedded platinum wires and placed into 20 ml vertical organ baths filled with Krebs–Henseleit-buffer (KHB) at 37° C and continuously bubbled with carbogen (95% O_2_/5% CO_2_). The other ending of the tissue strips was tied to a mechanoelectric transducer coupled to a bridge amplifier operating in differential (full bridge) mode (FORT10g/Transbridge TBM4M, both World Precision Instruments, USA). The output signal was sampled at a rate of 100/s, low-pass filtered with 2 Hz using a PowerLab8/32 (ADInstruments, Bella Vista, Australia) and then stored for further processing with LabChart (LabchartPro edition, Version 7.3.1, ADInstruments, Australia) and Excel 2010 (Microsoft, USA).

### Experimental protocol

At the very beginning of each experiment, a pre-strain of 3 mN was applied to the muscle strips which were then allowed to equilibrate for 45 min. Potassium concentration was then increased by 80 mM for 5 min using KCl (1.5 M stock solution), then restored by flushing with regular KHB and samples were again given 15 min rest. Subsequently, EFS was applied using a Grass S8 stimulator (Grass Technologies, USA) as 10-s-trains with 80 Volts, 10 Hz and 1 ms pulse duration. Trains were applied repeatedly every 100 s. When at least three uniform tissue responses had been elicited, TTX (1 µM) was added to the organ bath and further EFS-trains were applied until there were at least 3 uniform responses again. If spontaneous phasic activity was strong and interfered with EFS-responses, EFS trains were repeated as necessary until clearly distinguishable EFS-responses apart from spontaneous contractions were seen. After 10 min of equilibration without EFS, the CCh was added cumulatively to give a geometric series from 10 nM to 100 µM. Time intervals between single concentration steps were at least 3 min and were extended until a stable tone was reached. After repeated flushing with KHB, gas bubbling was reduced to a minimum to avoid foaming and the medium within the organ bath was completely replaced with serum from patients or controls. Following another 15 min of equilibration, at least three EFS-trains with the abovementioned parameters were applied. Following three hours of serum incubation, EFS was repeated and TTX (1 µM) was added with ongoing application of EFS trains every 100 s. Subsequently, still with serum present in the organ bath, CCh was applied using the same protocol as prior to serum incubation, followed by repeated flushing with KBS. After 20 min, potassium was raised again by 80 mM as described initially and the experiment was terminated.

### Drugs and solutions

The preparation buffer contained NaCl 145 mM, KCl 4.5 mM, NaH_2_PO_4_ 1.2 mM, MgSO_4_ 1.2 mM, CaCl_2_ 1 mM, EDTA 0.025 mM, HEPES 5 mM. The KHB solution contained NaCl 112 mM, NaHCO_3_ 25 mM, KH_2_PO_4_ 1.2 mM, KCl 4.7 mM, MgCl_2_ 1.2 mM, CaCl_2_ 2.5 mM, glucose 11.5 mM. Tetrodotoxin citrate, CCh (carbamoylcholine chloride) and all above-mentioned salts were purchased from Sigma-Aldrich (St. Louis, MO, USA). All substances were dissolved in double distilled water and stored according to the distributor’s recommendations.

### Statistics and calculations

Values of the concentration required for achieving 50% of the maximum contraction (EC_50_) were calculated by fitting equations of the type “% Effect size = 100/(1 + EXP((EC_50_ − Concentration)/slope))” to the normalized concentration-effect-data using the Excel-solver-function for a minimizing the least-square-deviation by parallel variation of slope and EC_50_. Distribution of samples was assessed by Shapiro–Wilk-tests. Depending on the presence or absence of normal distribution, data for groups are reported either as arithmetic means ± standard deviation (SD) or as median values with interquartile range (IQR). For simple bivariate and for partial correlations, Spearman’s rank order method was used, yielding the correlation coefficient ρ. Pearson’s correlation coefficient was calculated for normally distributed samples. For pairs of repeated measurements, Student’s t-test for paired samples or Wilcoxon’s paired sample tests, depending on the distribution of the variable were calculated. For independent samples, the corresponding Student’s t-test or the Mann–Whithney test were applied. For categorical data, χ^2^-tests were used. Comparisons between more than two groups were made using an ANOVA on ranks (Kruskal–Wallis-test) with Dunn’s post test due to the absence of normality in at least one group of all comparisons made. The post test included an automatic correction for multiple comparisons. For all tests, p-values < 0.05 were considered to indicate significance. All calculations were done with MSExcel2010 and SigmaStat 3.5 (Systat software Inc, USA).

## Abbreviations

ADM: Musculus abductor digiti minimi
ANOVA: Analysis of variance
CCh: Carbachol
CINM: Critical illness neuromyopathy
CMAP: Compound muscle action potential
EDB: Musculus extensor digitorum brevis
EFS: Electric field stimulation
ENG: Electroneurography
ICC: Interstitial cells of Cajal
ICU: Intensive care unit
ICUA-AW: Intensive care unit acquired weakness
K^+^: Potassium ions
KHB: Krebs–Henseleit-buffer
MRC-SS: Muscle research council sum score
SMC: Smooth muscle cell
SNAP: Sensory nerve action potential
SOFA: Sequential organ failure assessment
TTX: Tetrodotoxin
